# The Impact of the Neutrophil-to-Lymphocyte Ratio on In-Hospital Outcomes in Patients With Acute ST-Segment Elevation Myocardial Infarction

**DOI:** 10.7759/cureus.54418

**Published:** 2024-02-18

**Authors:** Gürkan Karaca, Ahmet Ekmekci, Ali Kimiaei, Seyedehtina Safaei, Nihat Özer, Gülşah Tayyareci

**Affiliations:** 1 Cardiology, Bahçeşehir University, Istanbul, TUR; 2 Cardiology, Okan University, Istanbul, TUR; 3 Cardiology, Dr. Siyami Ersek Thoracic and Cardiovascular Surgery Educational Research Hospital, Istanbul, TUR

**Keywords:** in-hospital outcomes, primary percutaneous coronary intervention (pci), acute st-segment elevation myocardial infarction (stemi), neutrophil-to-lymphocyte ratio (nlr), coronary artery disease

## Abstract

Introduction

The neutrophil-to-lymphocyte ratio (NLR) is a significant predictor of cardiovascular diseases, influencing their progression and prognosis. The exact role of the NLR in acute ST-segment elevation myocardial infarction (STEMI) is unclear. We investigated the possible association between peak NLR values within the first three days after STEMI onset and in-hospital outcomes in patients undergoing primary percutaneous coronary intervention (PCI).

Methods

This retrospective study included 641 patients who were diagnosed with acute STEMI and treated with primary PCI for 18 months at Dr. Siyami Ersek Hospital. The NLR was calculated using the maximum values obtained during the first three days after admission. The patients were divided into quartiles according to their NLR values for further analysis of potential complications during and after hospitalization, up to a follow-up period of three months.

Results

Significant differences were found in factors such as age, body mass index (BMI), and length of hospital stay among these groups. Specifically, we found that in-hospital mortality rates were significantly higher in the Q4 group, and there were variations in target vessel revascularization (TVR) rates, major adverse cardiac events (MACE) rates, and other clinical outcomes. Some parameters, such as reinfarction rates and certain procedural outcomes, did not show significant differences among the groups. However, despite the differences, most of the patients achieved successful outcomes after PCI, with the best results in the low NLR group and the worst results in the high NLR group.

Conclusion

Higher NLR values were associated with a higher risk of unfavorable outcomes during hospitalization.

## Introduction

Coronary artery disease (CAD) is a complex and multifactorial condition that involves various pathways such as inflammation, oxidative stress, myocardial injury, apoptosis, neurohormonal activation, and hemodynamic stress [1]. Biomarkers that reflect these pathways can help in the diagnosis, management, and prognosis of CAD and its complications [1]. The discovery of new biomarkers can also improve the clinical decision-making process and provide new insights into the pathophysiology and prevention of CAD [2]. One of the biomarkers that has gained attention in recent years is the neutrophil-to-lymphocyte ratio (NLR), which is a simple and inexpensive measure of the balance between inflammation and immunity [3]. The NLR is calculated by dividing the number of neutrophils by the number of lymphocytes in the blood. Several studies have shown that the NLR is a strong predictor of adverse outcomes in both stable and unstable CAD, as well as in patients undergoing coronary revascularization procedures such as coronary artery bypass grafting (CABG) or percutaneous coronary intervention (PCI) [4,5]. However, the exact role of the NLR in acute ST-segment elevation myocardial infarction (STEMI), which is the most severe form of CAD, is still unclear. The aim of this study was to investigate the possible association between peak NLR values within the first three days after STEMI onset and in-hospital outcomes in patients treated with primary PCI.

## Materials and methods

Study population

This retrospective study was conducted at the Cardiology Department of Dr. Siyami Ersek Cardiovascular Training and Research Hospital for 18 months. We included 641 patients (544 males and 97 females) who presented with chest pain and were diagnosed with acute STEMI according to the criteria of the American College of Cardiology/European Society of Cardiology [6]. All patients underwent PCI within six hours of symptom onset. The diagnosis of acute myocardial infarction (MI) was based on the presence of at least two of the following criteria: chest pain lasting longer than 30 minutes; at least 1 mm ST-segment elevation in two contiguous leads on the ECG, a newly developed Q wave, or a newly developed left bundle branch block; creatine kinase enzyme levels rising to twice the normal level, or the MB form being more than 5% of the total. We excluded patients who were older than 80 years, had an infection within the last 15 days, had chronic autoimmune-inflammatory diseases or active cancer, had acute or chronic kidney failure, had hematological proliferative diseases, or had received steroid treatment or chemotherapy. The treatment protocol was determined according to current guidelines. The study protocol was approved by the hospital’s ethics committee.

Data collection and analysis of patient information

All patients' medical history, demographic characteristics, cardiovascular history (previous myocardial infarction, bypass or percutaneous intervention, stable angina pectoris, heart failure or hospitalization with unstable angina, accompanying other systemic diseases, etc.), and risk factors (smoking, hyperlipidemia, hypertension, family history, diabetes mellitus) were recorded, and pain-to-balloon and door-to-balloon times were noted, along with physical examinations. At the time of admission, blood samples were taken for biochemical parameters and blood counts. An ECG was performed to record the ST-elevated myocardial infarction’s location and arrhythmias. Clinical measurements were made to determine the heart rate, blood pressure, and Killip heart failure classifications. Global left ventricular systolic ejection fractions were measured using System V (Vingmed, Oslo, Norway), a GE echocardiography device, and a 2.5 MHz phased-array transducer according to the modified Simpson's method. After primary percutaneous coronary intervention in each patient, ECG reperfusion criteria (greater than 50% reduction in ST-segment elevation at the 90th minute) and the full medical treatment given in the following days were recorded. Patients' daily laboratory findings at the time of admission and within the next 72 hours were evaluated. An automatic blood cell counting device was utilized to determine total leukocyte, neutrophil, lymphocyte, and monocyte numbers. From the same blood samples evaluated, maximum NLR measurements were extracted from peak counts of both neutrophils and lymphocytes. In-hospital courses and outcomes were obtained through clinical and laboratory follow-ups with patients.

Biochemical assessments and complete blood count measurements

Upon admission of the patients, blood samples were collected into tubes with purple caps for the evaluation of leukocyte, neutrophil, and lymphocyte counts. The normal reference ranges for these specific cells are as follows: leukocytes between 4,500 and 11,000/mm^3^; neutrophils between 1,500 and 6,700/mm^3^; lymphocytes between 1,500 and 4,000/mm^3^.

Coronary angiography, primary angioplasty, and stent implantation procedures

All patients received a loading dose of 300 mg of acetylsalicylic acid (ASA) and 600 mg of clopidogrel before the procedure. Angiographic data, including vessel type, lesion localization, pain-to-balloon time, and door-to-balloon time, were recorded for each patient. Coronary angiography and angioplasty procedures were performed via the femoral artery. Following arterial puncture, if unfractionated heparin (UFH) had not been administered to all patients upon arrival at the hospital, a bolus of 10,000 IU of UFH was administered intravenously. The flow in the infarct-related artery was evaluated according to the TIMI (Thrombolysis in Myocardial Infarction) classification. Primary angioplasty (balloon angioplasty and/or stent implantation) was applied only to the infarct-related artery based on the lesion type. The success of the procedure in the acute phase was defined as achieving TIMI II-III flow in the infarct-related artery with residual narrowing below 50%. Following angioplasty, patients were monitored in the coronary intensive care unit. Patients were prescribed 100 mg of aspirin and 75 mg of clopidogrel twice daily, along with 1 mg/kg of subcutaneous enoxaparin. Glycoprotein IIb/IIIa inhibitor therapy was left to the operator's choice, while β-blockers, ACE inhibitors, and statin therapy were applied according to the American College of Cardiology/American Heart Association (ACC/AHA) guidelines.

In-hospital follow-up of patients

Insights regarding in-hospital outcomes have been garnered through clinical and laboratory follow-ups of patients in the intensive care unit and during subsequent stages. This information was also extracted from direct interviews conducted with the patients. In addition to this, we continually monitored these individuals over an extended period while documenting their data.

Statistical analysis and interpretation

Statistical analyses were performed using NCSS (Number Cruncher Statistical System) 2007 and PASS (Power Analysis and Sample Size) 2008 Statistical Software (Utah, USA). In evaluating the study data, descriptive statistical methods (mean, standard deviation, median, frequency, and ratio) were used, as well as the one-way ANOVA test for comparing quantitative data with normally distributed parameters between groups and the Tukey HSD test for identifying the group causing the difference. In intergroup comparisons of parameters that do not show a normal distribution, the Kruskal-Wallis test was used, and the Mann-Whitney U test was used to determine the group causing the difference. Pearson’s Chi-Square test was used to compare qualitative data, and p<0.05 and p<0.01 were considered significant.

## Results

The study was conducted for 18 months at Dr. Siyami Ersek Cardiovascular Training and Research Hospital's Cardiology Clinic. A total of 641 cases were involved. The descriptive characteristics of the patients are provided in Table 1.

**Table 1:** Distribution of descriptive characteristics. BMI: Body mass index.

Variable	Description	Value
Age (years)	Mean ± SD	56.32±12.77
Gender		
- Female	n (%)	97 (15.1%)
- Male	n (%)	544 (84.9%)
Waist circumference (cm)	Mean ± SD	90.37±12.82
BMI (Kg/m^2^)	Mean ± SD	27.65±3.82
Length of hospitalization (days)	Mean ± SD (Median)	7.61±7.44 (6.0)
Family history	Yes; n (%)	200 (31.2%)
Hypertension	Yes; n (%)	261 (40.7%)
Diabetes	Yes; n (%)	122 (19.0%)
Hyperlipidemia	Yes; n (%)	151 (23.6%)
Smoking	Yes; n (%)	470 (73.3%)
Peripheral artery disease (PAD)	Yes; n (%)	16 (2.5%)
Stroke	Yes; n (%)	17 (2.7%)
Pre angina	Yes; n (%)	162 (25.3%)
Heart failure	Yes; n (%)	19 (3.0%)
Dialysis	Yes; n (%)	0 (0%)
Coronary artery bypass graft history (CABG)	Yes; n (%)	20 (3.1%)
History of percutaneous coronary intervention (PCI)	Yes; n (%)	71 (11.1%)
Myocardial infarction (MI)	Yes; n (%)	88 (13.7%)

Among the cohort, 97 were women (15.1%), 544 were men (84.9%); the average age was 56.32±12.77 years; the waist circumference was 90.37±12.82 cm; the body mass index was 27.65±12.82; and the average hospitalization duration was 7.61±7.44 days. In the risk factor assessment, 31.2% (n = 200) of the cases had a positive family history, 40.7% (n = 261) had hypertension, 19% (n = 122) had diabetes, 23.6% (n = 151) had hyperlipidemia, and 73.3% (n = 470) were smokers. In their medical history, 2.5% (n = 16) had peripheral artery disease, 2.7% (n = 17) had a stroke, 25.3% (n = 162) had pre-angina, 3% (n = 19) had heart failure, 3.1% (n = 20) had undergone previous CABG, 11.1% (n = 71) had percutaneous coronary intervention, and 13.7% (n = 88) had myocardial infarction. The total leukocyte (WBC), neutrophil, lymphocyte counts, and NLR of the patients on admission and on days 1, 2, and 3 are shown in Table 2.

**Table 2:** Leukocyte count, neutrophil count, lymphocyte count, and NLR of patients on admission and days 1, 2, and 3. WBC: White blood cell count; NLR: neutrophil-to-lymphocyte ratio.

Variable	Mean ± SD (median) at admission (day 1)	Mean ± SD (median) day 2	Mean ± SD (median) at day 3
WBC	12.46 ± 3.95 (12.00)	11.52 ± 3.50 (11.00)	10.27 ± 3.25 (9.80)
Neutrophil Count	9.65 ± 3.95 (9.10)	8.20 ± 3.42 (7.60)	6.80 ± 3.04 (6.10)
Lymphocyte Count	1.96 ± 1.18 (1.80)	2.25 ± 0.92 (2.20)	2.35 ± 0.93 (2.30)
NLR	6.57 ± 5.36 (5.41)	4.71 ± 4.40 (3.35)	3.57 ± 3.05 (2.57)
n	641	612	508

The NLR assessment involved utilizing the maximum values from four distinct tests. These ratios varied between 1.1 and 6.1, with an average of approximately 7.65±6.20 and a median value of 5.95. Quartile (percentile) ranges were defined as follows: the 25th percentile value was 3.95, the 50th percentile value was 5.95, and the 75th percentile value was 9.19. Subsequently, NLR classifications were determined, and assessments were grouped into four categories (Q1, Q2, Q3, Q4). The evaluation of descriptive characteristics by groups based on maximum NLR values is depicted in Table 3.

**Table 3:** Descriptive characteristics by groups based on maximum NLR values. BMI: body mass index; PAD: peripheral artery disease; CABG: coronary artery bypass grafting; PCI: percutaneous coronary intervention; MI: myocardial infarction.

Variable	Q1 (NLR<3.98) (n=160)	Q2 (NLR 3.98-5.95) (n=161)	Q3 (NLR 5.96-9.19) (n=160)	Q4 (NLR<9.19) (n=160)	P-value
Age (years); mean ± SD	55.21 ± 11.98	53.65 ± 13.73	56.37 ± 11.83	60.09 ± 12.68	0.001
Gender					0.934
- Female	25 (15.6%)	24 (14.9%)	22 (13.8%)	26 (16.3%)	
- Male	135 (84.4%)	137 (85.1%)	138 (86.3%)	134 (83.8%)	
Waist circumference (cm); mean ± SD	92.57 ± 11.65	89.90 ± 11.45	90.28 ± 13.02	88.73 ± 14.72	0.056
BMI (Kg/m^2^); mean ± SD	28.65 ± 4.54	27.57 ± 3.92	27.58 ± 4.17	26.78 ± 3.94	0.001
Length of hospitalization (days); mean ± SD (median)	6.69 ± 6.24 (6)	6.79 ± 4.58 (6)	6.91 ± 4.00 (6)	10.06 ± 11.78 (7)	0.001
Family history	54 (33.8%)	61 (37.9%)	47 (29.6%)	38 (23.8%)	0.043
Hypertension	60 (37.5%)	66 (41.0%)	69 (43.1%)	66 (41.3%)	0.778
Duration of hypertension (years); mean ± SD (median)	5.76 ± 5.08 (6)	5.82 ± 4.88 (6)	5.62 ± 5.25 (6)	6.67 ± 5.42 (8)	0.269
Diabetes	32 (20.0%)	26 (16.1%)	32 (20.0%)	32 (20.0%)	0.763
Hyperlipidemia	46 (28.8%)	33 (20.5%)	36 (22.5%)	36 (22.5%)	0.330
Smoking	130 (81.3%)	115 (71.4%)	119 (74.4%)	106 (66.3%)	0.022
PAD	6 (3.8%)	3 (1.9%)	5 (3.1%)	2 (1.3%)	0.461
Stroke	4 (2.5%)	4 (2.5%)	1 (0.6%)	8 (5.0%)	0.112
Pre angina	46 (28.8%)	46 (28.6%)	37 (23.1%)	33 (20.6%)	0.243
Heart failure	4 (2.5%)	4 (2.5%)	7 (4.4%)	4 (2.5%)	0.688
CABG	4 (2.5%)	7 (4.3%)	4 (2.5%)	5 (3.1%)	0.751
PCI	19 (11.9%)	17 (10.6%)	16 (10.0%)	19 (11.9%)	0.932
MI	19 (11.9%)	27 (16.8%)	21 (13.1%)	21 (13.1%)	0.611

A statistically significant age difference was observed among the groups (p<0.01). Specifically, the average age of the Q4 group was significantly higher than that of the Q1, Q2, and Q3 groups (p = 0.003; p = 0.001; p = 0.042; p<0.05), while no statistically significant age difference was detected among the other groups (p > 0.05). Contrary to this, no statistically significant differences were found between gender distributions, waist circumference measurements, hypertension prevalence rates and durations, diabetes, hyperlipidemia, PAD, stroke, pre-angina, heart failure, CABG, PCI, and MI prevalence rates. Regarding the body mass index measurements, a statistically significant difference was observed among the groups (p<0.01). Specifically, the body mass index of the Q1 group was significantly higher than that of the Q4 group (p = 0.001; p<0.01), while no statistically significant difference was found in the body mass indexes of the remaining groups (p > 0.05). Furthermore, our analysis revealed a statistically significant difference in hospitalization durations among the groups (p<0.01). Specifically, the Q4 group had a significantly longer average hospitalization duration compared to the Q1, Q2, and Q3 groups (p = 0.001; p = 0.001; p = 0.003; p<0.01), while no statistically significant difference was found in hospitalization durations among the other groups (p > 0.05). In terms of family history prevalence rates, we found a statistically significant difference between the groups (p<0.05), with the Q2 group having a significantly higher rate. Additionally, there was a statistically significant difference in smoking usage rates (p<0.05), with the Q1 group showing significantly higher rates. The analysis of pre-hospital parameters according to maximum NLR groups is detailed in Table 4.

**Table 4:** Assessment of pre-hospital parameters according to maximum NLR groups. Killip: Killip Classification; Anterior MI: anterior myocardial infarction; eGFR: estimated glomerular filtration rate; CK-MB: creatine kinase-MB; LDL: low-density lipoprotein; HDL: high-density lipoprotein; ASA: aspirin

Variable	Q1 (NLR<3.98) (n=160)	Q2 (NLR 3.98-5.95) (n=161)	Q3 (NLR 5.96-9.19) (n=160)	Q4 (NLR<9.19) (n=160)	P-value
Door to balloon	32.21 ± 8.10	34.08 ± 12.42	33.49 ± 12.61	33.34 ± 8.13	0.452
Pain to balloon	164.82 ± 2.03	172.78 ± 1.83	189.80 ± 1.79	180.72 ± 1.79	0.220
Killip; n (%)					0.001
- Class 1	159 (99.4%)	160 (99.4%)	152 (95.0%)	128 (80.0%)	
- Class 2	1 (0.6%)	1 (0.6%)	1 (0.6%)	6 (3.8%)	
- Class 3	0 (0%)	0 (0%)	3 (1.9%)	8 (5.0%)	
- Class 4	0 (0%)	0 (0%)	4 (2.5%)	18 (11.3%)	
Systolic blood pressure	127.80 ± 21.42	124.71 ± 21.28	124.99 ± 23.42	122.10 ± 31.28	0.234
Diastolic blood pressure	75.23 ± 14.21	73.56 ± 13.73	79.48 ± 65.33	72.41 ± 18.58	0.298
Heart rate	75.57 ± 10.80	78.04 ± 11.78	78.95 ± 12.00	84.25 ± 17.07	0.001
Anterior MI; n (%)					0.338
- Yes	66 (41.3%)	75 (46.6%)	82 (51.3%)	77 (48.1%)	
First creatinine	0.86 ± 0.23	0.83 ± 0.28	0.92 ± 0.31	1.01 ± 0.44	0.001
eGFR; (median)	117.76 ± 37.99 (116.5)	127.40 ± 57.01 (122.0)	108.02 ± 37.87 (106.0)	96.88 ± 51.91 (89.5)	0.001
Peak CK-MB	95.48 ± 2.05	109.34 ± 2.13	150.31 ± 2.19	193.37 ± 2.11	0.001
Total cholesterol	199.62 ± 49.63	192.06 ± 41.81	187.43 ± 42.11	181.22 ± 46.28	0.003
LDL	126.31 ± 38.01	122.75 ± 35.48	117.71 ± 37.49	112.46 ± 37.33	0.006
HDL	38.25 ± 9.94	36.68 ± 8.98	40.50 ± 11.98	40.85 ± 11.90	0.001
Triglyceride	148.80 ± 1.63	141.94 ± 1.77	133.04 ± 1.67	123.14 ± 1.69	0.009
First sugar; n (%)					0.688
- ≤ 105	13 (14.0%)	13 (13.7%)	12 (12.9%)	8 (8.8%)	
- > 105	80 (86.0%)	82 (86.3%)	81 (87.1%)	83 (91.2%)	
HbA1c; n (%)					0.784
- 4.8-5.9	54 (57.4%)	49 (51.6%)	54 (58.1%)	49 (53.8%)	
- >5.9	40 (42.6%)	46 (48.4%)	39 (41.9%)	42 (46.2%)	
Anemia; n (%)	15 (9.4%)	21 (13.0%)	25 (15.6%)	31 (19.4%)	0.074
WBC	10.48±2.97	11.70±2.91	12.70±3.20	14.98±74.93	0.001
Beta-blocker; n (%)	19 (11.9%)	23 (14.3%)	23 (14.4%)	23 (14.4%)	0.892
ACE inhibitor/ARB; n (%)	32 (20.0%)	35 (21.7%)	39 (24.4%)	47 (29.6%)	0.206
Spironolactone; n (%)	0 (0%)	0 (0%)	1 (0.6%)	0 (0%)	0.390
Statin; n (%)	16 (10.0%)	10 (6.2%)	13 (8.1%)	13 (8.2%)	0.672
ASA; n (%)	24 (15.0%)	23 (14.3%)	24 (15.0%)	33 (20.6%)	0.380
Clopidogrel; n (%)	2 (1.3%)	1 (0.6%)	5 (3.1%)	4 (2.5%)	0.331
Clopidogrel loading before coming to the hospital	335.63 ± 97.35	352.17 ± 114.06	335.63 ± 97.35	337.50 ± 99.53	0.399

According to the groups, there is no statistically significant difference in the incidence of door-to-balloon and pain-to-balloon in all cases (p > 0.05). However, a statistically significant variation was found between the incidence of Killip class in cases according to the groups (p<0.01). In all groups, the incidence of Killip class 1 is high, and higher Killip classes were observed in Q3 and Q4 cases. Additionally, a statistically significant difference was found in heart rate measurements (p<0.01), with the Q4 group exhibiting significantly higher measurements compared to the Q1, Q2, and Q3 groups (p=0.001; p=0.001; p=0.002; p<0.01). No statistically significant difference was detected in heart rate measurements among the remaining groups (p > 0.05).

No statistically significant difference was observed in the systolic and diastolic blood pressure measurements (p > 0.05) or the incidence of anterior MI (p > 0.05) among the cases according to the groups. In contrast, initial creatinine measurements exhibited a significant difference among the groups (p<0.01). Specifically, the Q4 group had significantly higher initial creatinine measurements compared to the other groups (p = 0.001; p = 0.001; p = 0.042; p<0.05), while no statistically significant difference was found in the initial creatinine measurements among the remaining groups (p > 0.05).

Furthermore, eGFR measurements displayed a significant difference among the groups (p<0.01). The eGFR measurements of the Q4 group were significantly lower than those of the Q1, Q2, and Q3 groups (p = 0.001; p = 0.001; p = 0.001; p<0.01). Additionally, the eGFR measurements of the Q3 group were significantly lower than those of the Q1 and Q2 groups (p = 0.030; p = 0.003; p<0.05). Nevertheless, no statistically significant difference was found in the eGFR measurements between the Q1 and Q2 groups (p = 0.348; p > 0.05).

Moreover, peak CK-MB measurements exhibited a significant difference among the cases (p<0.01). The peak CK-MB measurements of the Q4 group were significantly higher than those of the Q1, Q2, and Q3 groups (p = 0.001; p = 0.001; p = 0.015; p<0.05). Additionally, the Q3 group's peak CK-MB measurements were significantly higher than those of the Q1 and Q2 groups (p = 0.001; p = 0.001; p<0.01). However, no statistical difference was found between the peak CK-MB measurements of the Q1 and Q2 groups (p = 0.368; p > 0.05).

Total cholesterol measurements displayed a significant difference among the groups (p<0.01). Specifically, the total cholesterol measurements of the Q1 group cases were significantly higher than those of the Q4 group (p = 0.002; p<0.01), while no statistically significant difference was observed in total cholesterol measurements among the other groups (p > 0.05). Similarly, low-density lipoprotein (LDL) measurements exhibited a significant difference among the groups (p<0.01). The LDL measurements of the Q1 group were significantly higher than those of the Q4 group (p = 0.005; p<0.01), while no significant difference was found in LDL measurements among the other groups (p > 0.05).

High-density lipoprotein (HDL) measurements also showed a significant difference among the groups (p<0.01). Specifically, the HDL measurements of the Q2 group were significantly lower than those of the Q3 and Q4 groups (p = 0.010; p = 0.003; p<0.05), whereas no statistical significance was found in HDL measurements among the other groups (p > 0.05). Triglyceride measurements exhibited a significant difference among the groups (p<0.01). The triglyceride measurements of the Q1 group cases were significantly higher than those of the Q4 group (p = 0.008; p<0.01), while no significant difference was found in triglyceride measurements among the other groups (p > 0.05).

In contrast, initial sugar and HbA1c levels did not show statistical significance between the groups (p > 0.05). Furthermore, a significant difference was observed in WBC measurements among the groups (p<0.01). Specifically, in pairwise comparisons, the Q1 group had the lowest average WBC measurement, while the Q4 group had the highest. No statistically significant difference was found in WBC measurements among the other groups (p > 0.05). Lastly, no significant differences were found in the rates of anemia as well as the usage of beta-blockers, ACE inhibitors, angiotensin receptor blockers, spironolactone, statins, aspirin (ASA), and clopidogrel (p > 0.05). The analysis of in-hospital parameters according to maximum NLR groups is detailed in Table 5.

**Table 5:** In-hospital parameters according to maximum NLR​​​​​​​ groups. TVR: Target vessel revascularization; MACE: major adverse cardiac events; CRP: C-reactive protein; EF: ejection fraction; AF: atrial fibrillation; AV: atrioventricular; IABP: intra-aortic balloon pump; TIMI: thrombolysis in myocardial infarction; LMCA: left main coronary artery; LAD: left anterior descending coronary artery; C.X.: circumflex coronary artery; RCA: right coronary artery; PTCA: percutaneous transluminal coronary angioplasty; STENT: coronary stent; PTCA+STENT: percutaneous transluminal coronary angioplasty with stent.

In-hospital parameters	Q1 (NLR<3.98) (n=160)	Q2 (NLR 3.98-5.95) (n=161)	Q3 (NLR 5.96-9.19) (n=160)	Q4 (NLR<9.19) (n=160)	p-value
Death	0 (0%)	2 (1.2%)	4 (2.5%)	15 (9.4%)	0.001
Reinfarction	3 (1.9%)	4 (2.5%)	3 (1.9%)	8 (5.0%)	0.273
TVR	2 (1.3%)	4 (2.5%)	1 (0.6%)	9 (5.6%)	0.021
MACE	3 (1.9%)	6 (3.8%)	7 (4.4%)	24 (15.0%)	0.001
Stroke	0 (0%)	0 (0%)	2 (1.3%)	0 (0%)	0.110
CRP	0 (0%)	3 (1.9%)	3 (1.9%)	30 (18.8%)	0.001
Dialysis	0 (0%)	0 (0%)	3 (1.9%)	2 (1.3%)	0.141
VT/VF	5 (3.1%)	13 (8.1%)	11 (6.9%)	39 (24.4%)	0.001
Heart Failure	0 (0%)	1 (0.6%)	2 (1.3%)	19 (11.9%)	0.001
Inotrope	0 (0%)	3 (1.9%)	8 (5.0%)	37 (23.1%)	0.001
Shock	0 (0%)	2 (1.3%)	8 (5.0%)	28 (17.5%)	0.001
IABP	0 (0%)	1 (0.6%)	4 (2.5%)	13 (8.1%)	0.001
EF; Mean ± SD	47.84±7.43	46.21±7.80	44.47±8.51	42.78±9.59	0.001
AF	1 (0.6%)	2 (1.2%)	3 (1.9%)	9 (5.6%)	0.014
AV block	2 (1.3%)	4 (2.5%)	6 (3.8%)	12 (7.5%)	0.021
Proximal Lesion	101 (63.1%)	109 (67.7%)	101 (63.1%)	112 (70.0%)	0.470
Temporary Pacemaker	1 (0.6%)	4 (2.5%)	2 (1.3%)	10 (6.3%)	0.008
GIS Bleeding	1 (0.6%)	0 (0%)	1 (0.6%)	5 (3.1%)	0.036
Groin Complication	6 (3.8%)	5 (3.1%)	6 (3.8%)	7 (4.4%)	0.949
Acute Thrombosis	3 (1.9%)	4 (2.5%)	3 (1.3%)	3 (1.3%)	0.973
Sub-Acute Thrombosis	3 (1.9%)	2 (1.2%)	1 (0.6%)	8 (5.0%)	0.037
Late Thrombosis	0 (0%)	0 (0%)	3 (2.7%)	0 (0%)	0.031
Transfusion	2 (1.3%)	0 (0%)	2 (1.3%)	9 (5.7%)	0.002
Post-mechanical	0 (0%)	2 (1.2%)	0 (0%)	0 (0%)	0.113
ARTERY					0.416
LMCA	0 (0%)	1 (0.6%)	0 (0%)	1 (0.6%)	
LAD	67 (41.9%)	76 (47.2%)	82 (51.3%)	78 (48.8%)	
C.X.	32 (20.0%)	26 (16.1%)	22 (13.8%)	22 (13.8%)	
RCA	61 (38.1%)	56 (34.8%)	56 (35.0%)	58 (36.3%)	
Saphenous	0 (0%)	0 (0%)	0 (0%)	1 (0.6%)	
STROKE	0 (0%)	2 (1.2%)	0 (0%)	0 (0%)	
Number of Vessels					0.593
1 vessel	89 (55.6%)	83 (51.6%)	86 (53.8%)	75 (46.9%)	
2 vessels	48 (30.0%)	54 (33.5%)	44 (27.5%)	54 (33.8%)	
3 vessels	23 (14.4%)	24 (14.9%)	29 (18.1%)	31 (19.4%)	
4 vessels	0 (0%)	0 (0%)	1 (0.6%)	0 (0%)	
Pre-PCI-TIMI					0.313
1	155 (96.9%)	160 (99.4%)	158 (98.8%)	159 (99.4%)	
2	4 (2.5%)	1 (0.6%)	2 (1.3%)	0 (0%)	
3	1 (0.6%)	0 (0%)	0 (0%)	1 (0.6%)	
Pos-PCI-TIMI					0.001
1	0 (0%)	5 (3.1%)	5 (3.1%)	8 (5.0%)	
2	4 (2.5%)	4 (2.5%)	5 (3.1%)	16 (10.0%)	
3	156 (97.5%)	152 (94.4%)	150 (93.8%)	136 (85.0%)	
Procedure					0.415
PTCA	26 (16.3%)	32 (19.9%)	38 (23.8%)	35 (22.3%)	
STENT	37 (23.1%)	35 (21.7%)	25 (15.6%)	26 (16.6%)	
PTCA+STENT	97 (60.6%)	94 (58.4%)	97 (60.6%)	96 (61.1%)	
Conclusion					0.001
Successful	157 (98.1%)	155 (96.9%)	154 (96.9%)	143 (89.4%)	
Unsuccessful	3 (1.9%)	5 (3.1%)	5 (3.1%)	17 (10.6%)	

Statistically significant differences were evident across various clinical outcomes among the groups in our study. In-hospital mortality rates exhibited a significant difference (p<0.01), with a notably higher mortality rate observed in Q4 cases. However, no statistically significant differences were observed in the rates of reinfarction, stroke, dialysis, proximal lesion, groin complication, or post-mechanical occurrence between the groups (p > 0.05).

Significant differences were also noted in the rates of target vessel revascularization (TVR) occurrence (p<0.05), with a higher rate of TVR occurrence observed in Q4 cases. Furthermore, major adverse cardiac events (MACE) occurrence rates displayed a significant difference among the groups (p<0.01), with a higher incidence of MACE in the Q4 group. Additionally, a statistically significant difference was found between the groups in terms of C-reactive protein (CRP) elevation occurrence rates (p<0.01), with a higher rate of CRP elevation observed in Q4 cases.

In addition to that, the rates of ventricular tachycardia/fibrillation occurrence exhibited a significant difference between the groups (p<0.01), with a higher incidence in the Q4 group. Moreover, a statistically highly significant difference was found between the groups in the rates of heart failure occurrence and positive inotropic drug use (p<0.01, p<0.01). Heart failure and positive inotropic drug use were significantly higher in Q4 cases. Similarly, the rates of shock occurrence and intra-aortic balloon pump (IABP) use exhibited a significant difference between the groups (p<0.01), with Q4 cases showing significantly higher rates.

Furthermore, significant differences were observed in mean ejection fraction (EF) values among the groups (p<0.01). In pairwise comparisons, Q1 cases displayed higher EF measurements than Q3 and Q4 cases (p = 0.001; p = 0.002; p<0.01), and the EF measurements of Q2 cases were also significantly higher than those of Q4 cases (p = 0.002; p<0.01). The rates of atrial fibrillation (AF), atrioventricular (AV) block, and temporary pacemaker requirement occurrence showed significant differences among the groups (p<0.05, p<0.05, p<0.01), with the Q4 group having significantly higher rates.

Additionally, there was a statistically significant difference in the rates of gastrointestinal bleeding occurrence when evaluating complications (p<0.05), with Q4 cases experiencing significantly more gastrointestinal bleeding. While no statistically significant difference was observed in the rates of acute thrombosis occurrence (p>0.05), the rates of subacute thrombosis and late thrombosis occurrence displayed significant differences (p<0.05). Subacute thrombosis occurred at a higher rate in Q4 cases, whereas late thrombosis was only observed in Q3 cases.

Transfusion requirements also exhibited a statistically significant difference among the groups (p<0.01), with Q4 cases requiring significantly more transfusions. Conversely, there were no statistically significant differences in the rates of artery involvement, vessel number, and procedure occurrence (p>0.05). Moreover, pre-procedure TIMI Coronary Grade Flow scores did not display statistically significant differences (p > 0.05), but post-procedure TIMI scores exhibited a significant difference (p < 0.01). Specifically, post-procedure TIMI 1 and 2 scores were higher in Q4 cases, while TIMI 3 scores were higher in Q1 cases. Lastly, there was a statistically significant difference in the success outcomes of cases among the groups (p<0.01). Successful outcomes were predominantly achieved, with the highest success rate observed in Q1 cases and the lowest in Q4 cases. The survival analysis based on NLR quartiles is presented in Table 6.

**Table 6:** Survival results based on NLR quartiles. NLR: Neutrophil-to-lymphocyte ratio

NLR	n	Ex	Live	Survival Rate	Average survival time	Median survival time
Q1	160	0	160	100%	40.125 ± 5.99	-
Q2	161	2	159	98.8%	50.351 ± 0.456	7.0
Q3	160	4	156	97.5%	35.142 ± 0.423	6.0
Q4	160	15	145	90.6%	51.114 ±1 1.49	39.0

In the first quarter, no fatalities were recorded, with an average survival duration of 40.12±5.99 days. Moving into the second quarter, two deaths occurred, yet the average survival time extended to 50.35±0.4 days. Transitioning to the third quarter, four deaths were observed, correlating with an average survival time of 35.14±0.42 days. However, the fourth quarter saw a notable increase in mortality, with 15 deaths recorded, although the average survival time remained relatively high at 51.11±11.49 days. 

There was no statistically significant difference found in the three-month survival rates when we evaluated the NLR ratios using the log-rank test (p < 0.05). Overall, patients with higher NLR levels had higher rates of death, repeat procedures, and major complications than patients with lower NLR levels. They also had lower rates of successful PCI and normal blood flow in the arteries than patients with lower NLR levels. Patients with intermediate NLR levels had intermediate outcomes between the low and high NLR groups. There were no significant differences in some outcomes, such as reinfarction rates and procedural details, among the four groups. Patients with lower NLR levels had better outcomes than patients with higher NLR levels. They had lower rates of death, repeat procedures, and major complications than patients with higher NLR levels. They also had higher rates of successful PCI and normal blood flow in the arteries than patients with higher NLR levels.

## Discussion

Many studies have demonstrated that the total leukocyte count is related to major cardiovascular events after acute MI [7,8]. For example, some studies have established a correlation between neutrophil count and MI expansion, post-infarction heart failure development, impairment of epicardial and microvascular perfusion, and post-MI mortality [7,9-12]. However, information on subgroups remains limited. The effect of the NLR on in-hospital and subsequent cardiovascular events after acute MI is not well understood. Kyne et al. found a strong association between total leukocyte count and neutrophilia and the development of heart failure after acute MI, but they did not examine the NLR variable [7]. Núñez et al. found that the NLR was a better predictor of mortality than the peak total leukocyte count in patients with acute ST-elevation MI [13]. Our study also supports these findings. Núñez et al. included 470 consecutive patients and found higher mortality in the groups with high NLR [8]. They also found that a high NLR was correlated with female gender, hypertension, diabetes, high Killip’s class, low EF, new left bundle branch block (LBBB), atrial fibrillation (AF), renal insufficiency, ACE treatment, and blood glucose.

In a study with a large sample size, Salciccioli et al. found that a higher NLR was associated with an increased risk of 28-day mortality, ICU mortality, in-hospital mortality, and 1-year mortality. They also found that NLR values above 5 increased the risk of major adverse cardiovascular events (MACE) substantially [14].

Furthermore, Chebl et al., in a prospective study of 641 patients with sepsis, found that a higher NLR was associated with a higher risk of in-hospital mortality, heart failure, shock development, renal insufficiency development, and atrial fibrillation development. They also found that NLR values above 8.9 increased the risk of in-hospital mortality significantly [15].

Moreover, Li et al. found that a higher NLR was associated with a lower occurrence of spontaneous reperfusion and a higher incidence of MACE, cardiac death, recurrent MI, target vessel revascularization, and stent thrombosis. They also found that NLR values above 6.5 increased the risk of MACE significantly [16].

We divided 641 patients (544 males and 97 females), mostly male, into four groups based on their NLR levels. We found that higher NLR levels were associated with more severe cardiovascular events during hospitalization and follow-up. The group with the highest NLR (Q4) also had higher rates of heart failure, shock, renal insufficiency, and AF development. The groups were similar in terms of gender, ACE usage, hypertension, diabetes, and blood glucose levels, which made them more comparable.

The TIMI study discovered a connection between high leukocyte counts and soaring risks of heart failure or shock development within one month following an acute MI [17]. Not only this, but the same research posited similar findings about increased thrombus and microvascular damage in relation to a higher leukocyte count. Furthermore, several studies have shown that inflammatory mediators contribute crucially to both heart failure and unsuccessful reperfusion during acute MI [18,19]. Our results support the idea that inflammation affects the development of heart failure or shock after MI. As the NLR increased, heart failure, shock development, and the need for inotropic and IABP use also increased.

Inflammation is a major factor in myocardial cell injury during acute MI [20]. Drugs such as statins and ACE inhibitors, which are commonly used in acute MI, have anti-inflammatory effects. However, in our study, there was no difference in the use of these drugs between the groups, which suggests that high leukocyte counts and NLR are independent predictors of adverse outcomes in hospitalized patients with acute MI [21,22].

Our study shows that the NLR is a useful marker for predicting in-hospital morbidity and mortality. However, we did not find a significant difference between the groups at three-month follow-up, although the mortality rate was higher in the Q4 group. We are continuing to follow up with all patients in this study for long-term outcomes.

This study had some limitations. Its retrospective nature introduces biases, and being a single center may limit generalizability. Additionally, unmeasured confounders could influence the findings, and the short three-month follow-up might underestimate NLR's long-term impact. Larger prospective studies with extended follow-up are necessary to validate these findings and assess their clinical implications.

## Conclusions

In conclusion, our study provides evidence for the role of inflammation in the acute phase of STEMI and the prognostic value of NLR in predicting in-hospital adverse events. We found that a higher NLR was associated with more severe cardiovascular events, including heart failure, shock, renal insufficiency, and AF. The small sample size, lack of multivariate analysis, and lack of long-term follow-up data are among the limitations of our study. The NLR could be a simple and inexpensive biomarker for the risk stratification and management of patients with acute STEMI. However, further studies are needed to confirm these findings and explore the effects of anti-inflammatory therapies on the outcomes of these patients.
